# The impact of ATP-binding cassette transporters on metabolic diseases

**DOI:** 10.1186/s12986-020-00478-4

**Published:** 2020-08-03

**Authors:** Zixiang Ye, Yifei Lu, Tao Wu

**Affiliations:** Center of Chinese Medical Therapy and Systems Biology, Institute of Interdisciplinary Integrative Medicine Research, Shanghai University of Traditional Chinese Medicine, Cailun Road 1200, Shanghai, 201203 China

**Keywords:** ATP binding cassette transporters, Metabolic diseases, Obesity, Atherosclerosis, T2DM, Tangier disease

## Abstract

Currently, many people worldwide suffer from metabolic diseases caused by heredity and external factors, such as diet. One of the symptoms of metabolic diseases is abnormal lipid metabolism. ATP binding cassette (ABC) transporters are one of the largest transport protein superfamilies that exist in nearly all living organisms and are mainly located on lipid-processing cells. ABC transporters have been confirmed to be closely related to the pathogenesis of diseases such as metabolic diseases, cancer and Alzheimer’s disease based on their transport abilities. Notably, the capability to transport lipids makes ABC transporters critical in metabolic diseases. In addition, gene polymorphism in ABC transporters has been reported to be a risk factor for metabolic diseases, and it has been reported that relevant miRNAs have significant roles in regulating ABC transporters. In this review, we integrate recent studies to examine the roles of ABC transporters in metabolic diseases and aim to build a network with ABC transporters as the core, linking their transport abilities with metabolic and other diseases.

## Introduction

Currently, metabolic diseases are common in many countries. These diseases include a wide range of aliments, such as obesity, atherosclerosis (AS), stroke, type 2 diabetes mellitus (T2DM) and Tangier disease (TD). Individuals usually have several metabolic diseases at the same time. For example, people with obesity often also have AS or T2DM. In this case, it is promising and crucial to find a strategy that can effectively treat several metabolic diseases simultaneously.

Metabolic diseases can be caused by heredity and external factors such as diet. One of the roots of metabolic diseases is abnormal lipid metabolism. Recently, adenosine triphosphate (ATP)-binding cassette (ABC) transporters have become a research hotspot in metabolic disease therapy due to its ability to regulate lipid metabolism.

ABC transporters are one of the largest transport protein superfamilies [1]. The family of ABC transporters has 49 members that are further divided into 7 subfamilies from A to G according to the sequence similarity and domain organization of the members [2, 3]. ABC transporters are transmembrane proteins that are widely expressed in nearly all organisms and are located on cells and organelles. In humans, ABC transporters are particularly expressed in cancer cells and lipid-processing cells, such as macrophages [4–6].

ABC transporters can be classified into importers and exporters [7]. The transport mechanism of ABC transporter is controlled by alternating the conformation of transmembrane domains (TMDs), through which the transporter switches between inward- and outward-facing states [1]. Additionally, an increasing number of novel mechanisms have been explored. For example, Bi Y. et al. [8] discovered a processive O antigen translocation mechanism in gram-negative pathogens. Qian H. et al. [9] identified lateral access for ABCA1-mediated lipid export.

ABC transporters play a significant role in regulating the import and export of substances across plasma membranes. Driven by ATP, ABC transporters are able to carry a variety of substances across membranes such as small inorganic or organic molecules, sterol, metal ions, polypeptides and proteins [10, 11]. One of the applications of ABC transporters is to regulate lipid metabolism. Specifically, ABC transporters participate in cholesterol uptake, biosynthesis and storage to maintain cholesterol homeostasis [5]. ABC transporters facilitate the formation of high-density lipoprotein-cholesterol (HDL-C). In addition, ABC transporters are negatively correlated with fasting blood glucose, total cholesterol (TC), low-density lipoprotein-cholesterol (LDL-C) and triacylglycerol levels [12]. Since abnormal lipid metabolism is associated with the pathogenesis of metabolic diseases, ABC transporters participate in the inhibition of metabolic diseases, including AS, hypoalphalipoproteinemia (HA), coronary artery disease (CAD) and TD [13, 14].

ABC transporters have also received attention because of their effects on inducing multidrug resistance (MDR), especially in cancer conditions in which ABC transporters can reduce the efficacy of anticancer drugs [15]. In this case, ABC transporters are also regarded as members of detoxification families, such as P450 monooxygenases, glutathione-S-transferases, and carboxylesterases [16]. Additionally, ABC transporters also link metabolic diseases with neoplasms. Metabolic diseases promote tumors. For example, it has been reported that metabolic syndrome (MetS) is an obvious pathogenic factor for epithelial ovarian cancer [17]. The loss of cellular cholesterol homeostasis has been found frequently in prostate cancer cells [18]. Additionally, obesity has been confirmed to support the development and progression of melanoma and controlling body weight is effective for suppressing melanoma [19, 20]. Apart from reducing the expression of tumor markers and the precision of radiotherapy, excess adipose tissue is able to alter the pharmacokinetics of chemotherapeutic drugs. Obesity-associated adipokines, leptin and resistin, can upregulate ABCB1 (P-glycoprotein, P-gp). ABCB1 is part of the MDR family and facilitates the efflux of chemotherapeutic drugs from cancer cells. The efflux of drugs causes drug accumulation in plasma and consequently induces toxicity to organs [21]. Moreover, AS is correlated with cancer. It has been reported that up to 30% of cancer patient deaths are related to cardiovascular diseases [22]. Lectin-like oxidized low-density lipoprotein receptor-1 (LOX-1) is activated in AS. LOX-1 is a marker of AS and is induced by oxidized low-density lipoproteins (oxLDL). Since ABC transporters can decrease oxLDL levels, ABC transporters are likely to be impaired in AS. LOX-1 is capable of inducing adhesion molecules, proinflammatory signaling pathways and proangiogenic proteins such as NF-κB [23]. NF-κB can facilitate the production of reactive oxygen species (ROS), which then leads to lipid peroxidation and DNA damage that ultimately stimulate the progression of AS and cancer.

Overall, ABC transporters have a close relationship with metabolic diseases. In addition, their ability to cause MDR and facilitate metabolic diseases associated with cancer make ABC transporters a focus in cancer treatment. In this manner, an overall understanding of ABC transporters is key to treating cancer patients with metabolic diseases. In this review, we elucidated the structure and transport mechanism of ABC transporters. There are several ABC transporters that have a relationship with metabolic diseases. Additionally, we introduced a treatment strategy focusing on ABC transporters, including related microRNAs (miRNAs) and available drugs. The present review aims to make a contribution to building a network with ABC transporters as the core, linking transport functions with metabolic and other diseases.

## Structure of ABC transporters

Typically, ABC transporters consist of a combination of two TMDs and two cytoplasmic nucleotide-binding domains (NBDs). TMDs are variable in sequence and mechanism while NBDs are highly conserved and have characteristic amino acid motifs such as Walker A and Walker B [3, 24, 25]. Walker A and Walker B are key identifiers of ABC proteins [6]. TMDs and NBDs interact with each other closely to transport substances and hydrolyze ATP, respectively [24].

The numbers of TMDs and NBDs differ in different organisms. In prokaryotes, ABC transporters have all four of these domains, while in eukaryotes, the number of domains is diverse. Specifically, some proteins have all four domains, which are organized as a single polypeptide named full-transporter (FT). Other proteins have a single TMD and NBD and are called half-transporters (HTs), which need to homo or heterodimerize to be functional. Furthermore, there is an ABC2 structure with two NBDs and a single structure with only one TMD or NBD [3, 6, 11].

### The Transmembrane domain: a porter of substances

Two TMDs are intertwined with 4–6 transmembrane α-helices to form a cavity that is involved in substrate binding, recognition, transportation and making contacts with NBDs [4, 16, 24]. TMDs need to undergo conformational changes, and the degree of opening is diverse during transportation but has no close relationship with the size of the substance [1]. It has been reported that the location of pathogenic variations likely causes a shift in the pivot-like residue movement in TMDs, which weakens ATP binding and the interactions on the membrane surface [26]. In addition, several ABC transporters including ABCA1 and ABCC6 have an additional TMD (TMD0) at the amino-terminal extension region of the protein [4, 27]. TMD0 is approximately 250 amino acids long and is highly hydrophobic, with five transmembrane helices. This extra domain is important for normal functions and its absence can make the ABC protein unstable [24].

### The nucleotide-binding domain: the engine of ABC transporters

NBDs bind and hydrolyze ATP to supply energy for TMD conformational changes. This action is initiated by the presence of substrates [24, 28]. ATP is bound in the nucleotide binding site that is formed by an end-to-end connection of two NBDs [4].

### The substrate-binding protein: the initiator of ABC transporters

The substance is delivered by substrate-binding proteins (SBPs) that are fused to the TMDs to form one, two, or four substrate binding sites. SBPs consist of N- and C-terminal lobes connected by a hinge region. The lobes rotate facing each other to form a closed structure when SBPs bind a substrate. This form can productively trigger ATPase activity to initiate ABC transporters [4].

### ABC importer I and II

ABC importers can be further divided into importer I and II based on the numbers of transmembrane helices and the chemical nature of their substrates [10]. TMDs of type II importers including vitamin B12 and molybdate II transporters have more modest conformational changes but no specific substrate binding sites [4]. NBDs of type I importers typically have low ATPase activity and only hydrolyze ATP with high efficiency in the presence of a substrate-loaded SBP. In contrast, type II importers typically have high ATPase activity that is independent of substrate availability. Moreover, SBPs of type I importers undergo more significant conformational changes when binding to substrates than type II importers. Notably, many type I importers, such as maltose transporters, contain C-terminal regulatory domains that prevent ATP hydrolysis when the importer is bound to substrates. Interestingly, these two types of import systems can exist in a single transporter, such as the ribose transporter [7].

## Mechanisms of ABC transporters: an opening and closing transport gear

**Fig. 1:**
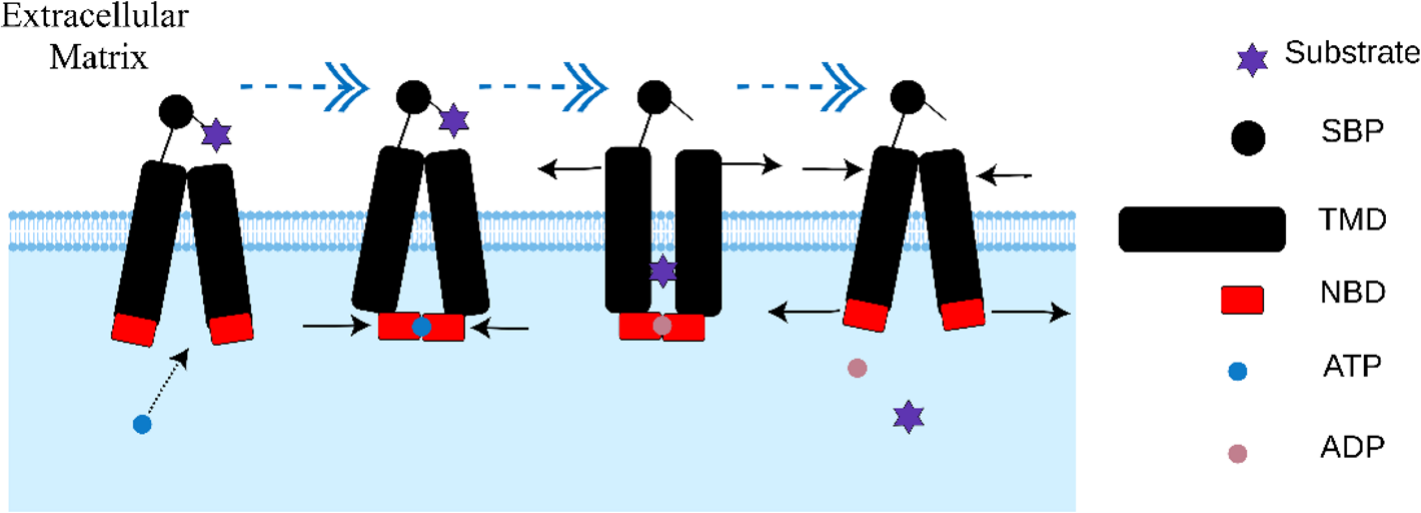
Mechanism of ABC importers. SBP binding to a substrate leads to NBD binding to ATP and a large-scale closing motion. The motion makes TMDs switch to the outward-facing state. The subsequent hydrolysis of ATP leads to the substrate crossing the lipid bilayer, and phosphate release initiates the return of the transporter to its initial state. Abbreviations: ABC, adenosine triphosphate-binding cassette; SBP, substrate-binding protein; NBD, nucleotide-binding domain; TMD, transmembrane domain

The transport function of ABC transporters is a circular process, as shown in Fig. 1. Taking the importer as an example, at the beginning, the transporter is in the inward-facing state. When periplasmic SBPs deliver a substrate to the TMDs, the NBDs bind and hydrolyze ATP, accompanied by a closing motion with sliding and rotation. This twisting motion is further propagated to the TMDs via the intracellular helices IH1 and IH2. In this manner, the TMDs are switched to the outward-facing state. Then, the substrate is delivered to the substrate binding site in the TMDs. The subsequent hydrolysis of ATP leads to substrate uptake across the lipid bilayer and phosphate release makes the transporter revert to the inward-facing state [29–31]. During this process, both ATP binding and SBP binding participate in maintaining the outward-facing state of the transporter [32].

## ABC transporter members

**Fig. 2:**
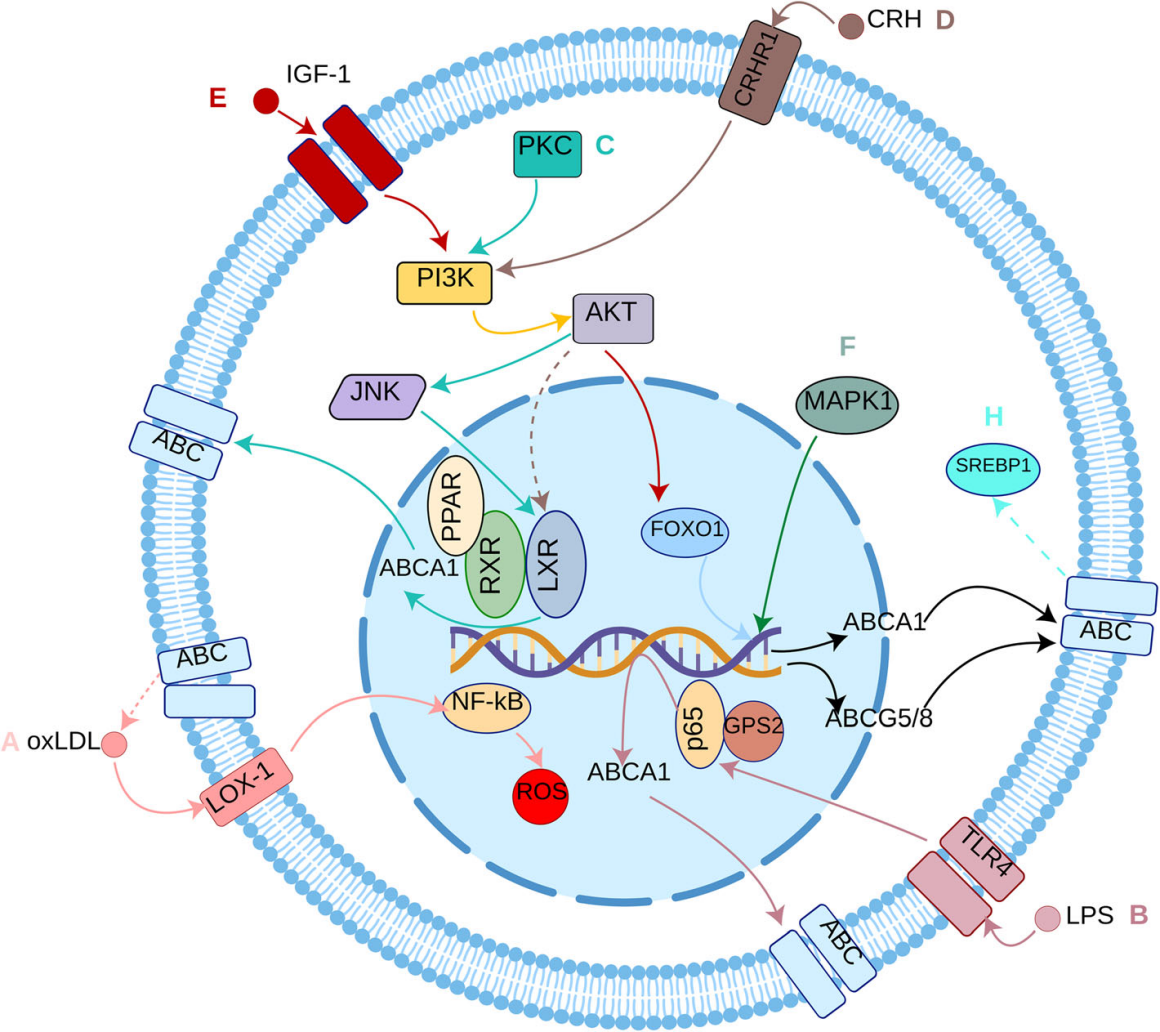
ABC transporter-related signaling pathways. A: Inhibition of ABC transporters can lead to an increase in oxLDL (shown by dotted line). oxLDL can cause inflammation through the LOX-1/NF-κB pathway to induce ROS production. B: LPS can induce ABCA1 via the TLR4/GPS2/NF-κB pathway to upregulate the gene expression of ABCA1. C: The PKC/PI3-K/Akt/JNK/LXR/RXR pathway is positively correlated with the expression of ABCA1. D: CRH can inhibit the activation of LXR (shown by dotted line) via the CRHR1/PI3-K/Akt pathway. E: IGF-1 can induce the PI3-K/Akt/FOXO1 pathway to activate FOXO1 which can facilitate the transcription of ABCA1 and ABCG5/8. F: MAPK1 can induce the expression of ABCA1. H: Expression of ABC transporters can inhibit the activation of SREBP (shown by dotted line), which can reduce lipogenesis. Abbreviation: ABC: Adenosine triphosphate-binding cassette; oxLDL: Oxidized low-density lipoproteins; LOX-1: Lectin-like oxidized low-density lipoprotein receptor-1; ROS: Reactive oxygen species; LPS: Lipopolysaccharides; TLR4: Toll-like receptor 4; GPS2: G protein pathway suppressor 2; PKC: Protein kinase C; PI3-K: Phosphatidylinositol 3-kinase; Akt: Protein kinase B; JNK: C-Jun N-terminal kinases; LXR: Liver X receptor; RXR: Retinoid-X-receptor; CRH: Corticotropin-releasing hormone; CRHR1: Corticotropin-releasing hormone receptor 1; IGF-1: Insulin-like growth factor 1; FOXO1: Forkhead box protein O1; MAPK1: Mitogen-activated protein kinase 1; SREBP: Sterol regulatory element binding protein; PPAR: peroxisome proliferator-activated receptor

Although all of the ABC transporters are capable of transmembrane transport, their substrates are diverse. For example, ABCA1, ABCA2, ABCA7, ABCA8 and ABCG1 transport lipids, while ABCC1 and ABCG2 transport xenobiotics, especially anticancer drugs. Regarding metabolic diseases, ABCA1 has attracted the most attention compared with that of the other ABC transporters. Moreover, there are several signaling pathways that can modulate the expression of ABC transporters or can be regulated by ABC transporters (shown in Fig. 2). Therefore, a deep understanding of the relationship between ABC transporters and related signaling pathways is important for treating relevant diseases. Here, we summarize a number of ABC transporters and their respective biological significance.

### ABCA1: the hottest research topic in metabolic disease

ABCA1 is widely expressed, especially in the liver, gastrointestinal tract, adipose tissue and macrophages [33]. This protein is expressed on the plasma membrane [34].

ABCA1 participates in the initial step of reverse cholesterol transportation (RCT) by regulating the movement of excess cholesterol and phospholipids from peripheral tissues to the liver. ABCA1 is able to form modest nascent HDL particles by binding with lipid-poor or lipid-free apolipoprotein (apo) A-I and apoE [35, 36]. ABCA1-knockout mouse models have demonstrated that ABCA1 is critical in mediating lipid efflux, and HDL formation and homeostasis [37]. ABCA1 generally cooperates with ABCG1 and scavenger receptor-BI (SR-BI) to assemble HDL [33]. The contribution of hepatic and intestinal ABCA1 to the plasma HDL pool is 70–80% and 15–20%, respectively [38]. In cells, ABCA1 mediates lipid transport between the Golgi and cell membrane [39].

ABCA1 also plays an important role in responding to abnormal cholesterol metabolism-related conditions, such as inflammation. Cholesterol efflux is relevant to inflammation at the transcription level [40]. ABCA1 has been reported to exert anti-inflammatory effects by inhibiting the production of some inflammatory cytokines in macrophages [13]. Moreover, ABCA1-mediated cholesterol efflux can inhibit lipopolysaccharide (LPS)-induced inflammatory signaling [41].

The ABCA1 gene is located on chromosome 9q31.1 [42]. Several gene polymorphisms of ABCA1 have been studied [43]. For example, the R219K (rs2230806) polymorphism is located at the N-terminal extracellular loop of the ABCA1 protein. I883M (rs4149313) is correlated with a marked decrease in HDL-C and cholesterol efflux [36, 39]. Mohammad M.B. et al. [13] reported that the increased risk of HA, decreased HDL-C and increased TG, interleukin 6 (IL-6) and C-reactive protein (CRP) are correlated with the ABCA1–565 C/T gene polymorphism.

ABCA1 transcription can be regulated by obligate heterodimers of liver X receptors (LXRs) and retinoid X receptors (RXRs), bacterial endotoxins such as LPS and peroxisome proliferator-activated receptors (PPARs) [40, 44, 45]. LXR is a transcription factor for ABCA1 [46]. Unsaturated fatty acids can suppress ABCA1 expression post transcriptionally via LXR and can facilitate ABCA1 degradation. LPS activates ABCA1 by inducing the cooperation of NF-κB subunit p65 and the transcriptional coregulator G protein pathway suppressor 2 (GPS2) [40]. In addition, endocytosis is able to regulate ABCA1 expression, which is mediated by the ADP-ribosylation factor 6 (ARF6)-dependent pathway [34].

### ABCA2: a potential solution to Alzheimer’s disease

The ABCA2 protein has the highest homology with ABCA1 due to the duplication of ancestral genes during evolution. It is naturally expressed in brain tissue and blood cells including macrophages, monocytes and blood stem cells. Furthermore, ABCA2 is mainly located intracellularly on late endosomes, lysosomes, the trans Golgi and the endoplasmic reticulum (ER). ABCA2 is able to maintain the homeostasis of sterols, sphingolipids and cholesterol in macrophages and neurons [47]. ABCA2 transporters have been reported to be related to diseases such as cholesterolemia, cardiovascular disease, Alzheimer’s disease and cancer.

### ABCA3: a possible target in respiratory diseases

Mutations in the ABCA3 gene have been confirmed to result in fatal surfactant deficiency, particularly respiratory distress syndrome (RDS) in babies and childhood interstitial lung disease [48, 49].

### ABCA7: a bridge connecting cholesterol metabolism with the immune system

Exogenous ABCA7 mediates the biogenesis of HDL from cellular lipids and helical apo. Endogenous ABCA7 has a relationship with phagocytosis, which is controlled by sterol regulatory element binding protein 2 (SREBP-2). Thus, ABCA7 is assumed to be a key molecule that connects cholesterol homeostasis and the body defense system [50]. The absence of ABCA7 has a significant impact on natural killer T (NKT) cell development and activation. In addition, overactive NKT cells have been implicated in the development of AS, autoimmunity, rheumatoid arthritis, and several forms of allergies [51]. Furthermore, ABCA7 has been considered a promising target in Alzheimer’s disease [52].

### ABCA8

It has been reported that ABCA8 acts as a sinusoidal efflux transporter for cholesterol and taurocholate in the liver [53].

### ABCB6: a supporter of cytochrome P450

ABCB6 deficiency suppresses cytochrome P450 (CYP450) expression in mouse and human hepatocytes. Ablation of ABCB6 reprograms the hepatic metabolic profile to negatively regulate hepatic CYP expression, possibly as a homeostatic response to promote survival. CYP450s constitute a superfamily of monooxygenases that play key roles in the metabolism of endogenous and exogenous compounds [54].

### ABCC1: one of the initiators of multidrug resistance

ABCC1, also known as multidrug resistance protein 1 (MRP1), prevents xenobiotics such as drugs from entering tissues. In addition, ABCC1 transports physiological substances such as folic acid, bilirubin, vitamin B12, and many glutathione and glucuronide conjugates. ABCC1 also secretes a variety of mediators that regulate redox homeostasis, inflammation, and hormone secretion. Overexpression of ABCC1 has been found in a variety of cancers such as lung cancer, leukemia and breast cancer [55].

### ABCG1: a porter of intracellular cholesterol

ABCG1 mainly accumulates in cells [56]. ABCG1 is expressed in the ER, trans-Golgi network (TGN) and endosomal recycling compartment (ERC) [5].

ABCG1 deficiency leads to profound intracellular cholesterol accumulation in macrophages and hepatocytes [57, 58]. Furthermore, ABCG1 is able to sense and manage membrane trafficking [5]. ABCG1 is short-lived, and both proteasomal and lysosomal inhibitors can decrease its degradation [5]. It has been reported that ABCG1 deficiency is associated with mild glucocorticoid insufficiency in mice because cholesterol is the sole precursor for glucocorticoid synthesis [59].

### ABCG2: the initiator of multidrug resistance in breast Cancer and the blood-brain barrier

ABCG2, also known as breast cancer resistance protein (BCRP), is abundantly expressed in the small intestine and liver [60]. ABCG2 is capable of affecting stem cell biology and controlling the excretion of various drugs, phase II metabolites, and endogenous substrates such as uric acid, toxic xenobiotics, and heme [15]. ABCG2 can excrete protoporphyrin (PP) from hepatocytes into the bile canaliculi. Thus, the formation of erythropoietic protoporphyria (EPP) may be a consequence of decreased ABCG2 expression, which ultimately leads to severe liver damage [61]. ABCG2 on the blood-brain barrier (BBB) also prevents therapeutic molecules from entering the brain parenchyma. Bakhsheshian J. et al. [62] established a relatively simple protocol for imaging ABCG2 at the BBB by determining the bioluminescence image (BLI) of luciferase because ABCG2 can specifically transport D-luciferin on the apical membrane of capillary endothelial cells in the BBB.

## *micro*RNAs and ABC transporters: a promising strategy

Since people often develop multiple metabolic aliments simultaneously, it is desirable to have a therapy that can target several metabolic diseases at the same time. Considering that one of the roots of metabolic disease is abnormal ABC transporter-related lipid metabolism, microRNA (miRNA) therapy has been identified as a promising strategy to radically treat metabolic diseases. MiRNAs are able to regulate mRNA transcription and can affect multiple genes within a signaling pathway or target several pathways at the same time [63]. Thus, if the miRNAs are carefully chosen, it is likely that related mRNAs in disease conditions can be specifically altered. In other words, the miRNA strategy can make treatment more targeted and have fewer side effects and toxicity than conventional therapy. In addition, the miRNA strategy can also treat hereditary metabolic diseases. The small size of miRNAs also overcomes the low effect and specific delivery of traditional gene therapy which uses large DNA plasmids or viral vectors encoding a protein [64]. Recently, miRNAs have been potential candidates as therapeutics (miRNA mimics) or targets of therapeutics (anti-miRs) [65]. An increasing number of miRNA therapies have been developed or applied to treat metabolic diseases and cancers [66]. Thus, it is important to identify ABC transport-related miRNAs and understand their effects on signaling pathways and the expression of ABC proteins. In this section, we described several miRNAs and their effects on ABC transporters (Table 1.)

**Table 1:** Reported miRNA and their target ABC transporter

miRNA	Targeted ABC transporter	Effect	Consequence	Reference
miR-19b	ABCA1	↓	Aggravating AS	Lv YC et al., 2015 [67]
miR-20	ABCA1	↓	Aggravating AS	Liang B et al., 2017 [68]
miR-23a-5p	ABCA1&ABCG1	↓	Aggravating AS&AIS	Yang S et al., 2018 [69]
miR-28	ABCA1	↑	Attenuating AS	Liu J et al., 2016 [70]
miR-33	ABCA1&ABCG1	↓	Aggravating Inflammation	Niesor EJ et al., 2015 [71] Lai L et al., 2016 [72]
miR-101	ABCA1	↓	Aggravating NAFLD&AS	Zhang N et al., 2015 [73]

Abbreviations: *miR* microRNA; *ABC* adenosine triphosphate-binding cassette; *AS* atherosclerosis; *AIS* acute ischemic stroke; *NAFLD* non-alcoholic fatty liver disease

### miR-19b

miR-19b promotes lipid metabolism abnormalities by binding to the 3′-UTR of ABCA1 transporters. It has been reported that glucagon-like peptide-1 (GLP-1) can upregulate the expression of ABCA1 by suppressing miR-19b [74].

### miR-20

miR20 can regulate ABCA1 at the posttranscriptional level and consequently influence cholesterol efflux and AS. miR-20a/b can suppress ABCA1 by directly binding to ABCA1 mRNA with low binding free energy. It has been found that miR-20a/b can decrease cholesterol efflux and increase cholesterol levels in THP-1 cells and RAW 264.7 macrophage-derived foam cells. Therefore, miR-20 inhibition constitutes a new strategy for ABCA1-based treatment of AS [68].

### miR-23a-5p

ABCA1 and ABCG1 are novel targets of miR-23a-5p. Yang S et al. [69] reported that the level of miR-23a-5p was elevated in plasma and macrophages in AS. In acute ischemic stroke (AIS) patients, carotid atherosclerotic plaque progression could be worsened by the presence of miR-23a-5p. miR-23a-5p antagonist therapy significantly reduced foam cell formation and AS progression and promoted plaque stability.

### miR-28

miR-28 can induce ABCA1 expression in patients with unstable angina by inhibiting mitogen-activated protein kinase 1 (MAPK1) [70]. The function of miR-28 has a close relationship with its host gene, LIM domain lipoma-preferred partner (LPP), which facilitates smooth muscle cell migration in arterial injury and AS.

### miR-33

miR-33 has been reported to downregulate ABCA1 and ABCG1 expression [71]. It has been identified as an indirect regulator of innate immunity that mediates bidirectional crosstalk between lipid homeostasis and inflammation by directly targeting Toll-like receptor (TLR) pathway members and cytokines. miR-33 augments macrophage lipid rafts and enhances proinflammatory cytokine induction and NF-κB activation by LPS. This effect occurs through an ABCA1- and ABCG1-dependent mechanism and is reversible by interventions with raft cholesterol and ABC transporter-inducing LXR agonists [72].

### miR-101

miR-101 can repress ABCA1 expression to facilitate intracellular cholesterol retention under inflammatory conditions. Zhang N et al. [73] suggested that miR-101 promoted the development of nonalcoholic fatty liver disease (NAFLD) and vascular AS.

## ABC transporters and metabolic diseases: abnormal lipid metabolism

**Fig. 3:**
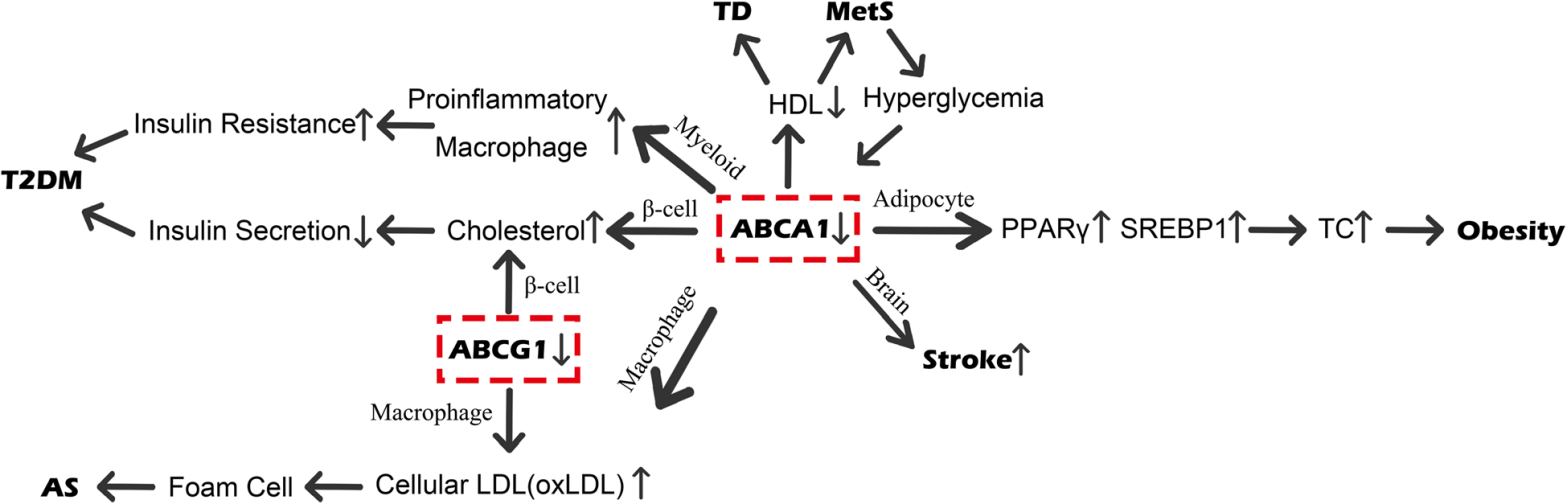
The relationship between ABC transporters and metabolic diseases. Abbreviation: ABC, adenosine triphosphate-binding cassette; PPARγ, peroxisome proliferator-activated receptor γ; SREBP1, sterol regulatory element binding protein1; TC, total cholesterol; HDL, high-density lipoprotein; TD, tangier disease; MetS, metabolic syndrome; LDL, low-density lipoprotein; oxLDL, oxidized low-density lipoprotein; AS, atherosclerosis; T2DM, type 2 diabetes mellitus

Since ABC transporters are responsible for mediating lipid homeostasis, they are closely related to metabolic diseases. Impaired ABC transporters can lead to excess free cholesterol which is toxic to adipocytes and macrophages. Toxicity can promote the unfolded protein response (UPR) and induce apoptosis via the ER [47]. Recently, the role of ABCA1 and ABCG1 in metabolic diseases has been the most studied (Fig. 3).

### Metabolic syndrome

MetS includes a variety of metabolic disorders and is characterized by increased waist circumference, hyperglycemia, hypertriglyceridemia, low HDL-C levels and hypertension. Chen WM et al. [12] suggested that MetS could be induced by impaired ABCA1 and that hyperglycemia further suppressed ABCA1 expression in macrophages via posttranscriptional regulation. At the same time, people with MetS were at high risk of developing T2DM and cardiovascular disease.

### Obesity

Obesity is a common metabolic disease. In obese adults, adipose tissues usually contain over 50% of the TC in the body [39]. Obesity is associated with reduced expression of ABCA1 in visceral adipose tissues [38]. Adipocyte ABCA1 is a key regulator of adipocyte lipogenesis and lipid accretion, likely due to increased cholesterol on adipose tissue membranes. ABCA1 can suppress the activity of the lipogenic transcription factors PPARγ and SREBP1 [75]. It has been confirmed in mouse models that a lack of adipocyte-specific ABCA1 leads to obesity. In addition, these mice without adipocyte-specific ABCA1 also showed decreased levels of the most active form of adiponectin and high-molecular-weight adiponectin [38]. Furthermore, GPS2 has been found to be downregulated in obese and T2DM subjects, providing additional evidence for the repression of ABCA1 in obesity and T2DM [40]. There are two single nucleotide polymorphisms (SNPs) of the ABCA1 gene, R219K and I883M, that have been found to have a close relationship with susceptibility to obesity. The SNP R219K increases susceptibility and phenotype severity, and of the SNP I883M plays a protective role [39].

### Atherosclerosis

The macrophage-mediated cholesterol uptake and metabolism is associated with the initiation and progression of AS in focal areas of the arterial subendothelial space [40]. The formation of lipid-laden macrophages, also known as foam cells, is the initial step of AS. Foam cell development is inhibited by macrophage expression of ABCA1 and ABCG1 [76]. Impaired macrophage ABCA1 and ABCG1 expression results in reduced cholesterol efflux and excess uptake of modified LDL, especially oxLDL, which further generates foam cells [46, 58]. The ABCA1 C69T polymorphism has been confirmed to be associated with increased AS risk [77]. Although RCT is a major mechanism by which HDL protects against AS, the atheroprotective effect of ABCA1 is not fully dependent on RCT [46, 78]. Interestingly, myeloid cell ABCA1 increases hepatic very-low-density lipoprotein (VLDL) TG secretion and plasma VLDL/LDL ratios, which reduces the atheroprotective effect and leads to a minimal increase in AS [79]. It has been reported that ABCG1 plays a role in promoting the progression of advanced atherosclerotic lesions by inducing the necrotic core of the atherosclerotic plaque [57]. Lipid-rich plaques can become unstable, leading to thrombosis, cardiac arrest and death. Davis W et al. [47] confirmed that ABCA2 can also reduce unstable lipid-rich plaques in macrophages. Cholesterol-induced toxicity can cause intracellular lipid-rich plaques, and ABCA2 can eliminate these plaques by modulating cholesterol trafficking from the late endosome/ lysosomal membrane compartment (LE/LY) to the ER in cells.

Several factors can affect the expression of ABCA1 and ABCG1. Sortilin is closely associated with hyperlipidemia and the risk of AS. Macrophage sortilin expression is upregulated by oxLDL in both concentration- and time-dependent manners. Sortilin can bind with ABCA1 and suppress macrophage ABCA1 expression by enhancing lysosomal degradation of ABCA1 [80]. Additionally, AS can also be caused by psychological stress factors such as corticotropin-releasing hormone (CRH). CRH can suppress ABCA1 and promote stress-related AS by activating CRH receptor 1 (CRHR1)-induced phosphorylation of protein kinase B (Akt). Phosphorylation of Akt is also induced by insulin and adiponectin to repress ABCA1 expression in human monocyte-derived macrophages. In addition, the phosphatidylinositol 3-kinase (PI3-K)/Akt signaling pathway suppresses the expression of both LXR and ABCA1 and can be activated by the cooperation of CRH and pregnancy-associated plasma protein-A (PAPP-A) [46].

AS is likely to cause CAD. CAD commonly manifests as reduced HDL-C levels, which are crucial factors in the morbidity and mortality of CAD patients [36]. The ABCA1 R219K polymorphism K allele shows a protective effect by increasing HDL-C levels, and its function has been reported to be independent of plasma lipid levels [36, 81]. The ABCA1 C-565 T promoter polymorphism is also a significant risk factor for the development and severity of CAD [82].

### Stroke

The ABCA1 gene is a key target of the transcription factor LXR. LXR activation has anti-inflammatory and neuroprotective effects in animal ischemic stroke models. Brain ABCA1 deficiency increases BBB leakage, white matter (WM)/axonal damage and functional deficits after stroke. Concomitant reduction of insulin-like growth factor 1 and upregulation of matrix metalloproteinase-9 may contribute to brain ABCA1 deficiency–induced BBB and WM/axonal damage in the ischemic brain [83]. In addition, Li Q et al. [58] reported that ABCG1 polymorphisms could attenuate ischemic stroke in a hypertriglyceridemic population with atherothrombotic stroke.

### Type 2 diabetes mellitus

T2DM is characterized by impaired insulin resistance, insulin secretion, and dysregulation of lipid and protein metabolism [42]. Impaired ABCA1 functions in pancreatic β-cells can cause the accumulation of cholesterol and a decrease in HDL-C, which leads to a reduction in insulin secretion and consequently reduces glucose tolerance, ultimately causing T2DM [84].

It has been reported that myeloid-specific ABCA1 gene deletion results in proinflammatory macrophages. Cytokines produced by macrophages in adipose tissue contribute to the development of insulin resistance [75]. However, there is a debate regarding whether the ABCA1 gene polymorphism is a genetic risk factor for T2DM [35, 85]. Insulin-like growth factor 1 (IGF-1) can mediate the stimulation of ABCA1 gene expression by inducing the PI3-K/Akt/Forkhead box protein O1 (FoxO1) pathway [86]. In addition, the expression of ABCA1 and miR-27a can be increased and decreased, respectively, via GLP-1. GLP-1 has been revealed to protect and improve pancreatic β-cell function against lipotoxicity which is considered one of the main causes of deterioration in β-cell function [87].

ABCG1 is essential for forming insulin granules, and ABCG1 deficiency inhibits insulin secretion by pancreatic β-cells. ABCG1, in addition to ABCA1 and oxysterol binding protein (OSBP), supports insulin granule formation [5]. Diabetes is usually accompanied by diabetic cardiovascular complications such as neointima formation because of endothelial progenitor cell (EPC) dysfunction. This dysfunction can be ameliorated by ABCG1 by promoting migration, tube formation and differentiation, and subsequent re-endothelialization of EPCs [88].

### Tangier disease

TD is characterized by nearly exhausted plasma HDL, increased TG levels, sterol deposition in tissue macrophages and increased susceptibility to coronary heart disease (CHD) [37, 89]. TD is caused by mutations in the ABCA1 gene. ABCA1-deficient HLCs fail to mediate lipid efflux or nascent HDL formation but have elevated TG secretion. In addition, it has been reported that TD patients have increased angiopoietin related protein 3 (ANGPTL3), which is an inhibitor of lipoprotein lipase (LPL), and influences HDL and TG metabolism [37]. TD patients also have hyporeactivity of blood platelets [16]. The absence of ABCA1 and low HDL levels reduce platelet reactivity by decreasing positive feedback loops, particularly thromboxane A2 (TXA2) production through a hematopoietic ABCA1-independent mechanism [90].

## Drug that mediate ABC transporters: ABC transporter antagonists and agonists

At present, an increasing number of drugs have been developed or are in development to treat ABC transporter-related metabolic diseases and cancer..

ABC transporters can lead to chemoresistance by facilitating drug efflux [91]. For example, overexpression of ABCB1 in human ovarian and breast cancer greatly suppresses the efficacy of chemotherapeutic drugs such as paclitaxel, vinca alkaloids and doxorubicin. Therefore, ABC transporter inhibitors have been considered an efficient way to restore tumor sensitivity to anticancer drugs. Recently, several man-made or natural ABC antagonists have been developed to treat cancer. Imatinib is a synthetic chemotherapeutic drug that is able to treat leukemia via down-regulating the mRNA and protein of ABCB1. In addition, it has been reported that natural products, including polyphenols, flavonoids and alkaloids, can reverse ABC transporter-related MDR [92]. For example, tetrandrine, a bisbenzylisoquinoline alkaloid isolated from *Stephania tetrandra* S. Moor, inhibits ABCC1 to reverse MDR in esophageal cancer [93].

Apart from treating cancer, ABC transporter antagonists and agonists are also utilized to alleviate metabolic diseases. These are ideal treatments for curing metabolic diseases because they can treat several metabolic diseases simultaneously. Here, we described several drugs and their mechanisms and effects on ABC proteins (Table 2). In summary, many of these drugs mediate mRNA expression and relevant signaling pathways to alter ABC transporters.

**Table 2:** Drugs to mediate ABC transporters

Drug	Target	Effect	Mechanism	Source	Reference
Statins	ABCA1	Reduce	↓PI3-K/Akt/FOXO1 & ↓TLR4/ NF-κB	Synthetic	Chen WM et al., 2016 [12]
Triptolide	ABCA1	Induce	↑LPS/TLR4/GPS2	Natural	Chen J et al., 2014 [41]
Cilostazol	ABCA1	Induce	↑LXR/ABCA1/SREBP-1	Synthetic	Jeon BH et al., 2015 [94]
H_2_S	ABCA1	Induce	↑PI3-K/Akt/ PPARα	Synthetic	Li D et al., 2016 [95]
Paeonol	ABCA1	Induce	↓Calpain-related pathway	Natural	Li X et al., 2015 [96]
Dgn	ABCA1	Induce	↓miR-19b expression	Natural	Lv YC et al., 2015 [67]
Hydrogen	ABCA1	Induce		Synthetic	Song G et al., 2015 [97]
Metformin	ABCG5& ABCG8	Induce	↑Ldlr ↑AMPK ↓ACLY	Synthetic	Molusky MM et al., 2018 [98]
CoQ10	ABCG1	Induce	↑Activator protein-1/miR-378/ABCG1	Natural	Wang D et al., 2014 [99]
Progesterone	ABCA2	Induce	↑mRNA expression	Synthetic& Natural	Davis W et al., 2018 [47]

Abbreviations: *ABC* adenosine triphosphate-binding cassette; *PI3-K* phosphatidylinositol 3-kinase; *Akt* protein kinase B; *FOXO1* forkhead box protein O1; *TLR4* Toll-like receptor 4; *LPS* lipopolysaccharides; *GPS2* G protein pathway suppressor 2; *LXR* liver X receptor; *SREBP-1* sterol regulatory element binding protein 1; *miR* microRNA; *H*_*2*_*S* hydrogen sulfide; *PPARα* peroxisome proliferator-activated receptor α; *Dgn* Diosgenin; *Ldlr* low density lipoprotein receptor; *AMPK* AMP-activated protein kinase; *ACLY* ATP citrate lyase; *CoQ10* Coenzyme Q10

### Statins

Stains have been reported to reduce ABCA1 expression. For example, atorvastatin can decrease Akt phosphorylation [12]. Thus, the PI3-K/Akt/FOXO1 pathway can be suppressed, which consequently suppresses the gene expression of ABCA1. In addition, statins can also downregulate ABCA1 mRNA by upregulating miR-33 levels. It is likely that miR-33 inhibits the TLR4/ NF-κB pathway to decrease the expression of ABCA1.

### Triptolide

Triptolide can regulate ABCA1 gene and protein expression to promote the expression of ABCA1, reducing the secretion of inflammatory factors and alleviating the lung pathological injury associated with LPS-induced acute lung injury (ALI) [41]. Thus, triptolide may mediate the expression of ABCA1 through the LPS/TLR4/GPS2 pathway to suppress inflammation.

### Cilostazol

Cilostazol, a phosphodiesterase 3, has been widely used to treat arterial disease and has additional beneficial effects on dyslipidemia. Cilostazol can ameliorate hepatic steatosis by increasing ABCA1 expression in hepatocytes [94]. The possible signaling pathway may be the LXR/ABCA1/SREBP-1 pathway in hepatocytes.

### Hydrogen sulfide

Li D et al. [95] indicated that hydrogen sulfide (H_2_S) had an antiatherosclerotic effect. NaHS (an exogenous H_2_S donor) treatment significantly increased the expression of ABCA1, apoA-I, and apoA-II to ameliorate intracellular lipid accumulation in HepG2 cells. H_2_S upregulates the expression of ABCA1 by promoting the nuclear translocation of PPARα, which is a transcription factor that regulates lipid metabolism [95]. The signaling pathway may be PI3-K/Akt/ PPARα.

### Paeonol

Paeonol, a potent antioxidant isolated from cortex moutan, exerts atheroprotective effects. Paeonol upregulates the protein stability of ABCA1 by inhibiting calpain activity and consequently affecting calpain-related pathways. Paeonol slows AS by repressing foam cell formation through heme oxygenase-1 (Ho-1)-dependent mediation of cholesterol efflux in macrophages. Furthermore, paeonol can modulate the expression of CD36 and ABCA1 in aortas [96].

### Diosgenin

Lv YC et al. [67] found that a structural analog of cholesterol, diosgenin (Dgn), could reduce blood fat levels and ameliorate atherosclerosis. Dgn enhanced ABCA1-dependent cholesterol efflux and inhibited aortic AS progression by suppressing macrophage miR-19b expression [67].

### Hydrogen

Hydrogen (dihydrogen [H_2_]) can suppress hypercholesterolemia and AS [97]. Specifically, H_2_ can decrease plasma LDL-C levels, activate ABCA1–dependent efflux and enhance HDL antiatherosclerotic functions in patients with potential MetS.

### Metformin

Molusky MM et al. [98] reported that metformin might upregulate macrophage RCT. The final steps of RCT are mediated by the sterol transporters, ABCG5 and ABCG8, which facilitate hepatobiliary transport of cholesterol. Metformin can provide cardiovascular benefits by increasing RCT and possibly enhancing low-density lipoprotein receptor *(*Ldlr*)* expression. Ldlr can activate AMP-activated protein kinase (AMPK) and can inhibit ATP citrate lyase (ACLY) to induce an increase in ABCG5/8, and ABCG5/8 can then facilitate the disposal of excess cholesterol by excretion into bile.

### Coenzyme Q10

Coenzyme Q10 (CoQ10) can increase macrophage RCT by regulating miRNA expression. Activator protein-1/miR-378/ABCG1 is a novel cascade by which CoQ10 facilitates macrophage cholesterol efflux. Both CoQ10 and miR-378 are promising candidates for AS prevention and treatment [99].

### Progesterone

Progesterone mimics the cellular physiological effects of ABCA2 in cells and elevates ABCA2 mRNA expression. Consequently, progesterone can alleviate cholesterol-induced toxicity in macrophages by modulating cholesterol trafficking from the LE/LY to the ER [47].

## Conclusions

Abnormal lipid metabolism commonly occurs in metabolic diseases, and ABC transporters have received increasing attention for their potential to treat several metabolic diseases simultaneously. ABC proteins can regulate lipid transport by an opening and closing transport cycle. In addition, the ability of ABC transporters to induce MDR and the promoting effect of metabolic diseases on cancer also highlight the significance of ABC proteins in cancer with concomitant metabolic disease.

In this review, we mainly focused on the relationship between ABC transporters and metabolic diseases. ABC transporters are able to improve cholesterol homeostasis by facilitating HDL-C assembly. Impairment of ABC transporters can lead to a variety of metabolic diseases, such as obesity, AS, T2DM and TD. The ABC transporter family can influence various signaling pathways to mediate the internal environment. For example, the expression of ABCA1 can suppress SREBP, which can repress lipogenesis. In addition, ABC transporters can also be controlled by several transcription factors. For example, LXR can induce the expression of ABCA1. FOXO1 can upregulate ABCA1 and ABCG5/8 expression at the same time. Among ABC transporters, ABCA1 and ABCG1 have received the most attention regarding metabolic diseases. Since other ABC transporters, such as ABCA2 have similar capabilities to control lipid transport, an extensive understanding of other family members might give us a broader scope to treat metabolic and related diseases. Moreover, MDR associated with ABC transporters in cancer has been intensively studied. However, MDR in metabolic diseases has not received much attention. We hope that further studies might focus on the influence of MDR on drugs to treat metabolic diseases to optimize ABC transporter-based therapy. In addition, it is important to clearly identify MDR-associated ABC transporter members to improve drug therapeutic effects.

ABC transporter-related miRNA therapy has been identified as a promising strategy to radically treat metabolic diseases. This therapy is expected to have a robust efficacy and excellent specificity. It is fundamental to have an overall understanding of the correlation with miRNAs and ABC proteins. Currently, several drugs have been developed or are in development to treat metabolic diseases by regulating signaling pathways and mRNAs. Additionally, metabolic diseases can promote the development of cancer. Thus, it is essential to selectively regulate the expression of different ABC transporters, especially in cancer patients with concomitant metabolic diseases.

## Data Availability

Not applicable.

## Abbreviations

ATP: Adenosine triphosphate
ABC: Adenosine triphosphate-binding cassette
TMDs: Transmembrane domains
MDR: Multidrug resistance
BBB: Blood-brain barrier
HDL-C: HDL cholesterol
TC: Total cholesterol
LDL-C: Low-density lipoprotein-cholesterol
AS: Atherosclerosis
HA: Hypoalphalipoproteinemia
CAD: Coronary artery disease
TD: Tangier disease
NBDs: Cytoplasmic nucleotide-binding domains
FT: Full-transporter
HT: Half-transporter
TMD0: Additional TMD
SBPs: Substrate-binding proteins
RCT: Reverse cholesterol transportation
apo: Apolipoprotein
SR-BI: Scavenger receptor-BI
T2DM: Type 2 diabetes mellitus
HLCs: Hepatocyte-like cells
TG: Triglyceride
LPS: Lipopolysaccharide
LXRs: Liver X receptors
RXR: Retinoid-X-receptor
PPARs: Peroxisome proliferator-activated receptors
NF-κB: Nuclear factor kappa-light-chain-enhancer of activated B cells
GPS2: G protein pathway suppressor 2
ARF6: ADP-ribosylation factor 6
IL-6: Interleukin 6
CRP: C-Reactive Protein
ER: Endoplasmic reticulum
RDS: Respiratory distress syndrome
NKT: Natural killer T
CYP450: Cytochrome P450
MRP1: Multidrug resistance protein 1
TGN: Trans-Golgi network
ERC: Endosomal recycling compartment
PP: Protoporphyria
EPP: Erythropoietic protoporphyria
BLI: Bioluminescence imagine
miRNAs: MicroRNAs
30-UTR: 30-untranslated region
SNPs: Single nucleotide polymorphisms
AIS: Acute ischemic stroke
MAP K1: Mitogen-activated protein kinase 1
LPP: Lipoma-preferred partner
TLR: Toll-like receptor
NAFLD: Non-alcoholic fatty liver disease
UPR: Unfolded protein response
MetS: Metabolic syndrome
oxLDL: Oxidized LDL
VLDL: Very-low-density lipoprotein
LE/LY: Late-endosome/ lysosomal membrane compartment
CRH: Corticotropin-releasing hormone
CRHR1: Corticotropin-releasing hormone receptor 1
Akt: Protein kinase B
PI3-K: Phosphatidylinositol 3-kinase
PAPP-A: Pregnancy-associated plasma protein-A
WM: White matter
IGF-1: Insulin-like growth factor 1
FoxO1: Forkhead box protein O1
GLP-1: Glucagon-like peptide-1
OSBP: Oxysterol binding protein
EPC: Endothelial progenitor cell
CHD: Coronary heart disease
ANGPTL3: Angiopoietin related protein 3
LPL: Lipoprotein lipase
TXA2: Thromboxane A2
ALI: Acute lung injury
H_2_S: Hydrogen sulfide
Ho-1: Heme oxygenase-1
Dgn: Diosgenin;
Ldlr: Lipoprotein receptor
AMPK: AMP-activated protein kinase
SREBP-2: sterol regulatory element binding protein 2
